# The value of routine blood work-up in clinical stratification and prognosis of patients with amyotrophic lateral sclerosis

**DOI:** 10.1007/s00415-023-12015-3

**Published:** 2023-10-06

**Authors:** Francesco Gentile, Alessio Maranzano, Federico Verde, Veronica Bettoni, Eleonora Colombo, Alberto Doretti, Marco Olivero, Francesco Scheveger, Claudia Colombrita, Ilaria Bulgarelli, Edoardo Gioele Spinelli, Erminio Torresani, Stefano Messina, Luca Maderna, Federica Agosta, Claudia Morelli, Massimo Filippi, Vincenzo Silani, Nicola Ticozzi

**Affiliations:** 1 Neurology Residency Program, Università degli Studi di Milano, Milan, Italy; 2 Department of Neurology, IRCCS Istituto Auxologico Italiano, P. Le Brescia 20, 20149 Milan, Italy; 3 Department of Pathophysiology and Transplantation, “Dino Ferrari” Center, Università degli Studi di Milano, Milan, Italy; 4 Department of Brain and Behavioral Sciences, IRCCS Mondino Foundation, Università degli Studi di Pavia, Pavia, Italy; 5 Department of Laboratory Medicine, Laboratory of Clinical Chemistry and Microbiology, IRCCS Istituto Auxologico Italiano, Milan, Italy; 6 Neurology Unit, IRCCS San Raffaele Scientific Institute, Milan, Italy; 7 Neuroimaging Research Unit, Division of Neuroscience, IRCCS San Raffaele Scientific Institute, Milan, Italy; 8 Vita-Salute San Raffaele University, Milan, Italy; 9 Neurorehabilitation Unit, IRCCS San Raffaele Scientific Institute, Milan, Italy; 10 Neurophysiology Service, IRCCS San Raffaele Scientific Institute, Milan, Italy

**Keywords:** Amyotrophic lateral sclerosis, Blood, Biomarkers, Survival

## Abstract

**Background:**

There is an unmet need in amyotrophic lateral sclerosis (ALS) to provide specific biomarkers for the disease. Due to their easy availability, we aimed to investigate whether routine blood parameters provide useful clues for phenotypic classification and disease prognosis.

**Methods:**

We analyzed a large inpatient cohort of 836 ALS patients who underwent deep phenotyping with evaluation of the clinical and neurophysiological burden of upper (UMN) and lower (LMN) motor neuron signs. Disability and progression rate were measured through the revised ALS Functional Rating Scale (ALSFRS-R) and its changes during time. Cox regression analysis was performed to assess survival associations.

**Results:**

Creatinine significantly correlated with LMN damage (*r* = 0.38), active (*r* = 0.18) and chronic (*r* = 0.24) denervation and baseline ALSFRS-R (*r* = 0.33). Creatine kinase (CK), alanine (ALT) and aspartate (AST) transaminases correlated with active (*r* = 0.35, *r* = 0.27, *r* = 0.24) and chronic (*r* = 0.37, *r* = 0.20, *r* = 0.19) denervation, while albumin and C-reactive protein significantly correlated with LMN score (*r* = 0.20 and *r* = 0.17). Disease progression rate showed correlations with chloride (*r* = −0.19) and potassium levels (*r* = −0.16). After adjustment for known prognostic factors, total protein [HR 0.70 (95% CI 0.57–0.86)], creatinine [HR 0.86 (95% CI 0.81–0.92)], chloride [HR 0.95 (95% CI 0.92–0.99)], lactate dehydrogenase [HR 0.99 (95% CI 0.99–0.99)], and AST [HR 1.02 (95% CI 1.01–1.02)] were independently associated with survival.

**Conclusions:**

Creatinine is a reliable biomarker for ALS, associated with clinical features, disability and survival. Markers of nutrition/inflammation may offer additional prognostic information and partially correlate with clinical features. AST and chloride could further assist in predicting progression rate and survival.

**Supplementary Information:**

The online version contains supplementary material available at 10.1007/s00415-023-12015-3.

## Introduction

Amyotrophic lateral sclerosis (ALS) is a neurodegenerative disorder characterized by predominant motor neuron loss in the brain and spinal cord, leading to progressive paralysis, bulbar dysfunction and respiratory failure within 3–5 years [1]. The diagnosis relies on clinical findings and neurophysiology, while blood tests, neuroimaging, and CSF studies are mainly employed to exclude alternative etiologies. In recent years, efforts to identify new reliable factors to be used for diagnosis and clinical trial design led to the discovery of new blood biomarkers, such as neurofilament light and phosphorylated heavy chains (NfL and pNfH, respectively), whose role in detection and risk stratification of ALS has been validated in several studies [2, 3]. However, application of Nf measurement in clinical practice may be hindered by the advanced technologies required to obtain reproducible results, currently limiting its availability in several ALS centers. Other studies looked for potential repurposing of common blood parameters, attracted by their easy availability and inexpensiveness. Among all, serum creatinine levels showed the strongest association with ALS, correlating with disease disability, severity and survival [4–6]. Markers of muscle loss, such as creatine kinase [4], and of inflammation, including serum albumin [6] and C-reactive protein [7], also showed significant correlations with disease survival, but more studies are needed to validate these findings.

In this study, we explored the potential association of a battery of blood parameters, measured during clinical practice, with disease features and prognosis in a large cohort of ALS patients. In particular, we determined their association with site of onset, markers of upper (UMN) and lower motor neuron (LMN) damage, disability, and survival.

## Materials and methods

### Patients and study design

A cohort of inpatients with ALS (*n* = 845) was recruited at the Department of Neurology of IRCCS Istituto Auxologico Italiano, Milan, Italy, between January 2005 and April 2022. The diagnosis was based on the revised El Escorial Criteria [8]. In all patients, routine blood tests were performed at time of first evaluation in our institution and included 33 blood parameters: complete blood count (white blood cells [WBC], red blood cells [RBC], hemoglobin [Hb], platelets [Plt], polymorphonucleate cells [PMN], lymphocytes [Ly], monocytes [Mo]), complete metabolic panel (alanine [ALT] and aspartate [AST] transaminases, gamma-glutamyl transferase [gGT], alkaline phosphatase [ALP], bilirubin, urea, creatinine [Crea], sodium [Na^+^], potassium [K^+^], chloride [Cl^−^], calcium [Ca^2+^], glucose, lactate dehydrogenase [LDH], urate, total protein, albumin, cholinesterase [CHE]), creatine kinase (CK), lipid profile (total cholesterol, HDL, LDL, triglycerides [TG]), inflammatory markers (erythrocyte sedimentation rate [ESR], C-reactive protein [CRP]), vitamin B12 and thyroid-stimulating hormone (TSH). Patients with chronic kidney disease (*n* = 6) as well as those showing an acute inflammatory state (*n* = 3) were excluded from the analysis. All blood exams were collected and measured in the same laboratory, to ensure homogeneity of the results.

Severity of UMN and LMN damage was evaluated using the Penn UMN score (PUMNS) [9], as well the MRC sum score (MRC-s) and a modified version of the LMN score [10], as previously described [11]. Furthermore, we measured the neurophysiological burden of LMN damage, using a semiquantitative score for active and chronic denervation (AD and CD, respectively) in affected muscles, that we recently validated [12].

Functional impairment was assessed through the ALSFRS-R scale (range 0–48) [13] and rate of progression (ΔALSFRS-R) was calculated according to the formula (48-ALSFRS-R)/disease duration at evaluation expressed in months. Survival was calculated from time of onset to death or tracheostomy. Patients were also screened for the hexanucleotide repeat expansion in the *C9orf72* gene, as previously described [14].

### Ethics

We received approval for this study from the Ethics Committee of Istituto Auxologico Italiano IRCCS (2021_05_18_04). Written informed consent for using anonymized clinical data for research purposes was obtained at the time of evaluation from all patients included in the analysis. This study conforms with the Declaration of Helsinki on human research.

### Data availability

The data that support the findings of this study have been published on Zenodo (https://zenodo.org/record/7642558) and are available upon reasonable request. The data are not publicly available due to privacy or ethical restrictions.

### Statistical analysis

Data are expressed in numbers (%) or median (range). Descriptive parameters were compared by the Chi-squared or Wilcoxon–Mann–Whitney tests, where appropriate, and adjusted for sex and age of onset. Spearman’s correlation test was performed for correlation analyses and a Bonferroni correction was applied for multiple testing. Cox regression analysis was used to evaluate the prognostic role of each blood parameter separately, adjusted for known prognostic factors (age of onset, time to first evaluation/diagnostic delay, *C9orf72* expansion). Those blood analytes associated with survival were then included in a new multivariate Cox model with backward conditional algorithm (cut-off set at < 0.05) to identify independent prognostic factors. Hazard ratios with their 95% confidence intervals (HR, 95% CI) were calculated. Creatinine values were multiplied by 10 to measure HR for each unit of 0.1 mg/dL. To assess significant changes in model accuracy according to inclusion/exclusion of blood parameters, the Akaike Information Criteria (AIC) was calculated and compared between the two models [15, 16]. Subsequently, the model was re-run after converting the continuous scale of significant blood parameters to an ordinal scale according to quartiles, to identify discrete HRs associated with specified cut-offs. Significant p-value for survival analysis was maintained at < 0.05. SPSS (SPSS Inc., USA) version 26 was used for all analyses.

## Results

### Cohort description

A total of 836 ALS patients were included in the study (Table 1). Median age of onset was 61 years (13–87), with male prevalence (527/836, 63.0%). A spinal onset was observed in 638 cases (76.3%), while 198 (23.7%) had a bulbar onset. A *C9orf72* repeat expansion was detected in 47 out of 765 tested patients (6.1%). Median disease duration at evaluation was 12.5 months (1.9–273) while median overall survival in our cohort was 48 months (interquartile range 30–94).

**Table 1:** Frequency of blood test abnormalities and differences according to site of onset in ALS patients

	*N*	Normal range	%Ab	Spinal onset	Bulbar onset	Adj *p*-value
Demographics						
Male sex	836			425/638 (66.6%)	102/198 (51.5%)	
Age of onset	836			61 (13–87)	64 (20–87)	
Time to evaluation	836			13 (1–503)	11 (2–86)	
*C9orf72* expansion	813			31/620 (5%)	17/193 (8.8%)	
ALSFRS-R	538			40 (10–48)	41 (16–47)	
Blood count						
Hb (g/dL)	836	13.8–18 (M) 11.9–16 (F)	20.6%	14.15 (8.4–19)	13.55 (9.5–17.5)	**0.04**
Mo (10^9^/L)	836	0.1–1.5	0%	0.5 (0.2–1.3)	0.5 (0.1–1)	**0.01**
Metabolic panel						
LDH (U/L)	831	< 250	59.0%	293 (72–662)	291 (116–545)	*ns*
CK (U/L)	835	< 190	44.8%	201 (16–3768)	109.5 (26–891)	**3.4e−08**
Tot proteins (g/dL)	834	6.5–8.5	35.1%*	6.7 (5.2–9.4)	6.75 (5.5–8.4)	*ns*
Glucose (mg/dL)	830	70–100	25.5%	92 (35–354)	89 (70–172)	*ns*
B12 (ng/L)	731	211–911	14.9%	443 (100–4000)	431 (85–4000)	*ns*
ALT (U/L)	836	< 40	14.2%	24 (4–127)	18 (6–118)	**1.8e−06**
AST (U/L)	836	< 40	7.8%	23 (4–112)	20.5 (9–97)	**0.02**
Urate (mg/dL)	823	2.0–7.0	5.5%	4.9 (1.2–10.3)	4.5 (1.5–10.6)	**0.018**
Creatinine (mg/dL)	832	0.4–1.4	1.8%*	0.7 (0.1–1.4)	0.8 (0.4–1.4)	**3.6e−07**
Lipid profile						
LDL (mg/dL)	824	< 115	62.0%	127 (22–277.2)	125 (32–240)	*ns*
Tot cholesterol (mg/dL)	821	< 200	51.2%	202 (109–397)	203 (110–331)	*ns*
HDL (mg/dL)	824	> 40	13.2%	53 (25–145)	57 (29–130)	*ns*
TG (mg/dL)	825	< 150	18.9%	106 (29–625)	92 (35–749)	*ns*
Inflammation						
ESR (mm/h)	828	< 15	28.4%	9 (2–120)	10.5 (2–67)	*ns*

Data presented as number (percentage) or median (range). For the sake of clarity, only parameters with a frequency abnormality of > 10% or showing a significant difference between patients with spinal vs bulbar onset were included. Significant* p*-values are reported in bold. For the complete table see Supplementary Table S2

*%Ab* percentage of patients with abnormal values; *ns* not significant

*Low levels in all

Data coverage was more than 95% for all blood parameters except for vitamin B12 and CRP levels, available in 731 (87%) and 576 (69%) patients, respectively. The most frequently altered blood tests at evaluation were those reflecting dyslipidemia (high LDL 61%, high CHO 51%, high TG 19%, low HDL 13%), followed by high LDH (59% of cases), high ESR (45%), high CK (44.5%) and low total protein levels (35%) (Table 1 and Supplementary Table S1).

### Spinal vs bulbar onset

We found significantly higher levels of CK (*p* = 3.4e−08), ALT (*p* = 1.8e−06) and AST (*p* = 0.02) in patients with spinal onset, while levels of serum creatinine were slightly increased in cases with bulbar-onset ALS (*p* = 3.6e−07; Table 1 and Supplementary Table S1). A significant difference also emerged for hemoglobin and absolute number of monocytes, whose levels appeared mildly higher in patients with spinal compared to those with bulbar onset (*p* = 0.04 and *p* = 0.01, respectively). No differences were observed for the remainder of blood analytes between the two groups.

### Markers of upper and lower motor neuron damage

**Fig. 1:**
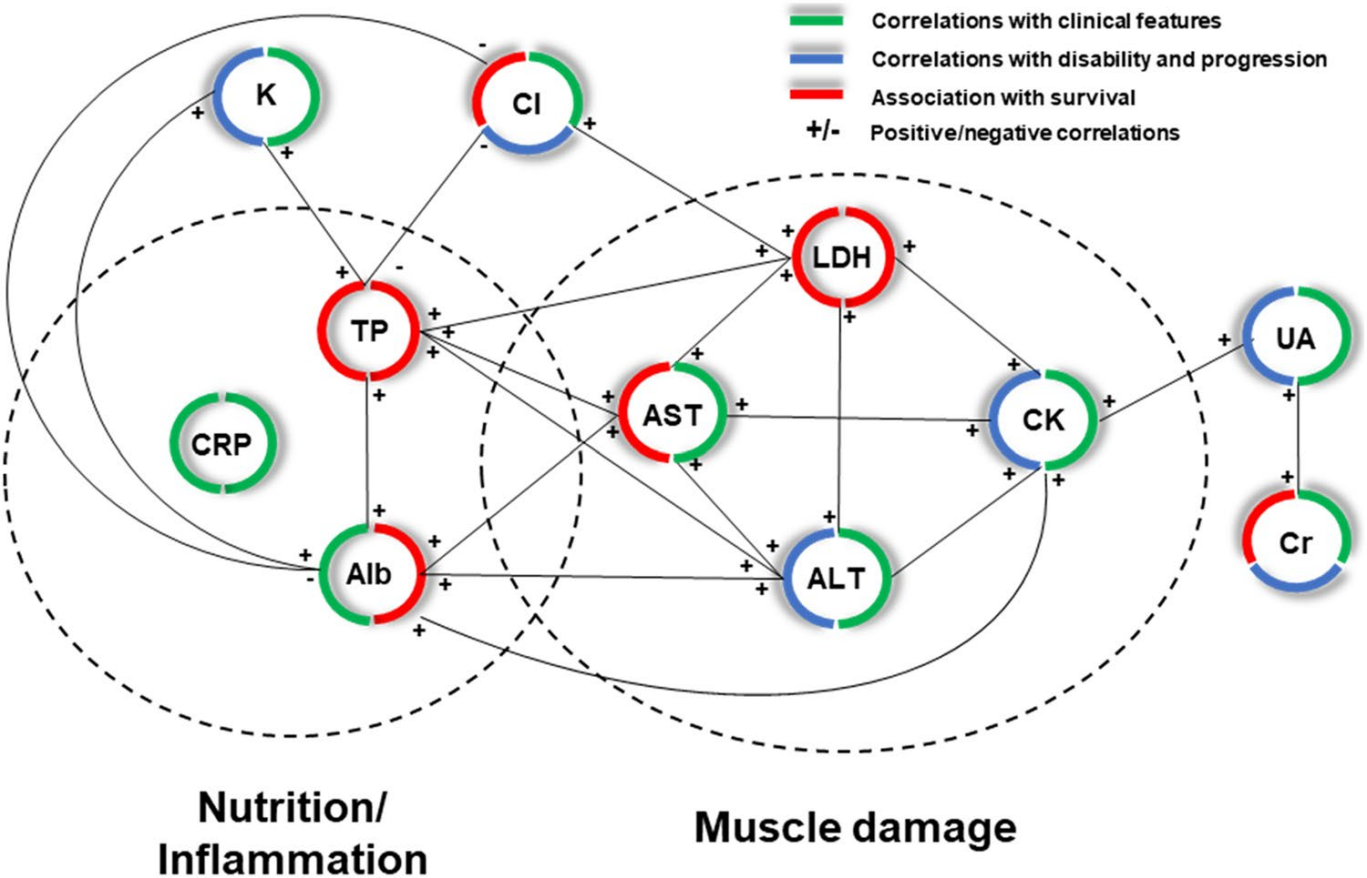
Summary chart of the correlation network of blood parameters associated with amyotrophic lateral sclerosis. The correlations with three clinical domains of ALS, namely clinical features (UMN/LMN signs), disability, and survival, were labelled with different colours (green, blue, and red, respectively). ± positive/negative correlations

Correlations between blood parameters and clinical/neurophysiological markers of UMN and LMN damage are reported in Table 2, Supplementary Table S2 and Fig. 1. Creatinine inversely correlated with both clinical and neurophysiological signs of LMN damage, namely MRC-s (*r* = 0.41, *p* = 1.9e−25) and LMN score (*r* = −0.38, *p* = 2.6e−25), as well as AD (*r* = −0.18, *p* = 1.8e−04) and CD (*r* = −0.24, *p* = 1.3e−08) scores. Significant, albeit weaker, correlations with both clinical scales were also found for albumin (MRC-s: *r* = 0.16, *p* = 0.006; LMN score: *r* = −0.16, *p* = 7.7e−04) and CRP (MRC−s: *r* = −0.21, *p* = 0.001; LMN score: *r* = 0.17, *p* = 0.02). Neurophysiological scores instead showed more consistent correlations with CK (AD: *r* = 0.35, *p* = 7.2e−19; CD: *r* = 0.37, *p* = 2.9e−21), followed by ALT (AD: *r* = 0.27, *p* = 9.6e−11; CD: *r* = 0.20, *p* = 2.1e−05) and AST (AD: *r* = 0.24, *p* = 1.5e−08; CD: *r* = 0.19, *p* = 5.1e−05). Cl^−^ showed significant correlations with both AD (*r* = −0.18, *p* = 4.6e−04) and CD (*r* = −0.19, *p* = 1e−04), while K^+^ appeared linked only to AD (*r* = −0.17, *p* = 0.001). PUMNS negatively correlated with CK (*r* = −0.31, *p* = 8.7e−17) and ALT (*r* = −0.18, *p* = 1.2e−05), while minor associations were identified with urate and glucose levels (Table 2).

**Table 2:** Correlations of blood tests with UMN and LMN signs

	UMN score		MRC score		LMN score		AD score		CD score	
	rho	(*p*)	rho	(*p*)	rho	(*p*)	rho	(*p*)	rho	(*p*)
Creatinine	0.05	ns	0.41	**2.0e − 25**	− 0.38	**2.6e − 25**	− 0.18	**1.8e − 04**	− 0.24	1.3e − 08
Albumin	− 0.02	ns	0.14	ns	− 0.2	**5.9e − 04**	− 0.02	ns	− 0.03	ns
CRP	− 0.01	ns	− 0.21	**1.1e − 03**	0.17	**2.0e − 02**	0.1	ns	0.12	ns
CK	− 0.31	**8.7e − 17**	− 0.15	**2.0e − 02**	0.09	ns	0.35	**7.1e − 19**	0.37	2.8e − 21
ALT	− 0.12	ns	− 0.21	**1.2e − 05**	0.11	ns	0.27	**9.6e − 11**	0.2	2.1e − 05
AST	− 0.18	**1.4e − 04**	− 0.11	ns	0.07	ns	0.24	**1.6e − 08**	0.19	5.1e − 05
Cl^−^	0.05	ns	0.06	ns	− 0.15	**7.1e − 03**	− 0.18	**4.6e − 04**	− 0.19	1.0e − 04
K^+^	− 0.05	ns	0.13	ns	− 0.12	ns	− 0.17	**1.2e − 03**	− 0.07	ns
Glucose	− 0.15	**4.5e − 03**	0.04	ns	− 0.04	ns	0.13	ns	0.07	ns
Urate	− 0.16	**1.5e − 03**	0.05	ns	− 0.07	ns	0.09	ns	0.06	ns

Correlations of routine blood parameters with UMN and LMN scores. Data for UMN and LMN scores were available in 776 patients, neurophysiological scores in 699 patients and the MRC sum score in 640 patients. All *p*-values are reported after Bonferroni correction (165 comparisons). Significant p-values are reported in bold

*AD* active denervation; *CD* chronic denervation; *LMN* lower motor neuron; *ns* not significant; *UMN* upper motor neuron

### Functional impairment and progression

Using the total ALSFRS-R at time of diagnosis, we observed significant positive correlations with creatinine (*r* = 0.33, *p* = 1.4e−12), CK (*r* = 0.18, *p* = 0.007) and K^+^ (*r* = 0.17, *p* = 0.01) levels (Supplementary Table S3, Fig. 1). Disease progression rate (ΔALSFRS-R) showed only a significant negative correlation with two electrolytes, K^+^ (*r* = −0.19, *p* = 0.001) and Cl^−^ (*r* = −0.16, *p* = 0.04), while no significant relationship was found for the other blood tests.

### Survival analysis

After correction for known prognostic factors (age of onset, time to first evaluation/diagnostic delay and *C9orf72* expansion), twelve blood parameters, namely AST (HR 1.012, 1.005–1.02, *p* = 0.001), ALT (HR 1.01, 1.004–1.016, *p* = 0.002), gGT (HR 1.003, 1–1.005, *p* = 0.022), LDH (HR 0.998, 0.997–0.999, *p* = 0.002), urea (HR 0.986, 0.974–0.998, *p* = 0.025), creatinine (HR 0.86 for every 0.1 mg/dL increase, 0.81–0.92, *p* < 0.001), urate (HR 0.88, 0.81–0.95, *p* = 0.001), total protein (HR 0.82, 0.69–0.98, *p* = 0.033), albumin (HR 0.74, 0.58–0.95, *p* = 0.018), K^+^ (HR 0.70, 0.53–0.92, *p* = 0.01), Cl^−^ (HR 0.95, 0.92–0.99, *p* = 0.005) and TSH (HR 0.89, 0.81–0.97, *p* = 0.01) were significantly associated with risk of mortality in our ALS cohort (Supplementary Table S4). In the next step, a multivariate Cox regression analysis, including known prognostic factors together with the above-mentioned twelve biochemical factors, identified total protein (HR 0.70, 0.57–0.86, *p* < 0.001), creatinine (HR 0.86 for every 0.1 mg/dL increase, 0.81–0.92, *p* < 0.001), Cl^−^ (HR 0.95, 0.92–0.99, *p* = 0.009), LDH (HR 0.998, 0.997–0.999, *p* = 0.001) and AST (HR 1.017, 1.009–1.024, *p* < 0.001) as independent factors associated with survival (Supplementary Table S5; Fig. 1). To assess the impact of blood parameters on survival prediction, we compared the Akaike Information Criteria (AIC) of two models: one that includes blood parameters (Model 1) and another that consists solely of known prognostic factors (Model 2). A lower AIC indicates a more accurate model. The difference in AIC values (ΔAIC) between the two models exceeded 10 (Model 1 AIC = 3815, Model 2 AIC = 3834, ΔAIC_Model 2-Model 1_ = 19). This strongly suggests that incorporating blood parameters into the composite survival model significantly improves its predictive accuracy. Notably, the exclusion of LDH from the analysis did not lead to a significant loss in model accuracy (ΔAIC < 2) (Table 3). To identify potential impactful cut-offs, we constructed subgroups for each blood variable according to quartiles. Using the highest quartile as reference, we observed that the risk of death increased by up to 2.5 times with lower levels of creatinine (≥ 0.9 mg/dL [ref] vs < 0.9 and ≥ 0.75 HR 1.69, 1.23–2.31, *p* = 0.001; vs < 0.75 and ≥ 0.6 HR 1.96, 1.46–2.62, *p* < 0.001; vs < 0.6 HR 2.53, 1.72–3.73, *p* < 0.001), up to 1.6 times with lower total protein levels (≥ 7.1 g/dL [ref] vs < 7.1 and ≥ 6.7 HR 0.81, 0.61–1.09, p = 0.161; vs < 6.7 and ≥ 6.3 HR 1.38, 1.01–1.89, p = 0.046; vs < 6.3 HR 1.62, 1.17–2.26, *p* = 0.004), up to 2.2 times with lower Cl^−^ levels (≥ 106 mmol/L [ref] vs < 106 and ≥ 104 HR 1.25, 0.94–1.67, *p* = 0.121; vs < 104 and ≥ 102 HR 2.2, 1.58–3.07, *p* < 0.001; vs < 102 HR 1.81, 1.32–2.48, *p* < 0.001), and up to 1.5 times with higher levels of AST (< 18 U/L vs ≥ 29 HR 1.34, 0.95–1.89, *p* = 0.091; ≥ 18 and < 23 vs ≥ 29 HR 1.52, 1.13–2.03, *p* = 0.005; ≥ 23 and < 29 vs ≥ 29 HR 1.38, 1.02–1.88, *p* = 0.039) (Table 3).

**Table 3:** Multivariate Cox regression analysis of blood analytes in ALS

	Model 1		Model 2		Akaike information criteria (AIC)
	HR (95% CI)	*p*	HR (95% CI)	*p*	
Age of onset	1.03 (1.02–1.05)	***p* < 0.001**	2.04 (1.82–2.29)	***p* < 0.001**	
*C9orf72 hre*	2.06 (1.39–3.06)	***p* < 0.001**	1.78 (1.22–2.6)	***p* < 0.003**	
Time to first evaluation	0.93 (0.92–0.94)	***p* < 0.001**	0.50 (0.45–0.56)	***p* < 0.001**	
AST (≥ 29 U/L)	1				AIC Model 1 = 3815
≥ 23 and < 29^a^	0.72 (0.53–0.98)	***p* = 0.039**			
≥ 18 and < 23^b^	0.66 (0.49–0.88)	***p* = 0.005**			AIC Model 2 = 3834
< 18 U/L	0.75 (0.53–1.06)	*p* = 0.105			
Creatinine (≥ 0.9 mg/dL)	1				ΔAIC = 19
< 0.9 and ≥ 0.75	1.69 (1.23–2.31)	***p* = 0.001**			
< 0.75 and ≥ 0.6	1.96 (1.46–2.62)	***p* < 0.001**			
< 0.6	2.53 (1.72–3.73)	***p* < 0.001**			
Total protein levels (≥ 7.1 g/dL)	1				
< 7.1 and ≥ 6.7	0.81 (0.61–1.09)	*p* = 0.161			
< 6.7 and ≥ 6.3	1.38 (1.01–1.89)	***p* = 0.046**			
< 6.3	1.62 (1.17–2.26)	***p* = 0.004**			
Cl^−^ (≥106 mmol/L)	1				
< 106 and ≥ 104	1.25 (0.94–1.67)	*p* = 0.121			
< 104 and ≥ 102	2.2 (1.58–3.07)	***p* < 0.001**			
< 102	1.81 (1.32–2.48)	***p* < 0.001**			

Prognostic role of routine blood parameters in ALS, categorized in quartiles. The lowest AIC, the better the model fitness. A difference of AIC of more than 10 is strongly supportive of better accuracy in survival prediction of model 1 including known prognostic factors and blood parameters compared to model 2 with only known prognostic factors. The AIC of the original model including LDH was 3816, very similar to model 1 with LDH excluded (ΔAIC < 2). Significant p-values are reported in bold

^a^Using this category as reference, when compared to ≥ 29 U/L: HR 1.38 (1.02–1.88), *p* = 0.039

^b^Using this category as reference, when compared to ≥ 29 U/L: HR 1.52 (1.13–2.03), *p* = 0.005

### Correlations between blood parameters

A thorough correlation analysis was performed among blood parameters to identify potential biases in our survival model due to highly correlated covariates which may overshadow independent associations with survival, due to a process known as multicollinearity [17]. A comprehensive summary of correlation coefficients and their *p*-values is reported in Supplementary Table S5 while Fig. 1 illustrates the relationships among the blood factors (AST, ALT, LDH, CK, creatinine, uric acid, total protein, albumin, CRP, K^+^, Cl^−^) which we found to be associated with ALS. Two potential clusters of parameters can be observed, grouped as markers of muscle damage (group 1) and nutrition/inflammation (group 2). In the first category, besides the expected high correlation between AST and ALT (*r* = 0.78, *p* = 1.4e−166), CK showed an association with AST (*r* = 0.54, *p* = 3.8e−62), ALT (*r* = 0.42, *p* = 2.7e−33) and LDH (*r* = 0.33, *p* = 7.4e−19), confirming that these analytes may serve as secondary markers of muscle damage. In the second group, total protein and albumin also displayed a strong correlation (*r* = 0.77, *p* = 2.2e−158), and both were significantly linked to AST, ALT, K^+^ and Cl^−^ (Fig. 1, Supplementary Table S5).

Due to the high correlations observed between AST/ALT and total protein/albumin, we cannot exclude that the independent association observed for AST and total protein over ALT and albumin, in spite of similar biological links, is due to this bias. For this reason, taking into account these two pairs of variables, we constructed two new multivariate survival models by excluding AST in the first and total protein levels in the second alternative model. Indeed, after exclusion of AST levels, ALT was independently associated with survival (HR 1.011, 1.005–1.017, *p* < 0.001) and the same was observed for albumin (HR 0.73, 0.55–0.96, *p* = 0.02) after the exclusion of total protein levels. Notably, all the other significant blood factors retained in the two models were the same observed in the first analysis (creatinine, Cl^−^ and LDH), supporting consistency and reproducibility of our approach.

## Discussion

In this referral-based cohort of 836 ALS patients, we showed that blood factors may be effectively repurposed for disease characterization and prognostic stratification (Fig. 1). Creatinine stood as the strongest blood biomarker in ALS, showing consistent correlations with clinical and neurophysiological signs of LMN damage, as well as with disability and survival, with a proportional increased risk of death (HR up to 2.5) with lower levels. Decreasing total protein levels and albumin were also independently associated with shorter survival (HR up to 1.6), with albumin also correlating with clinical LMN features. AST and Cl^−^ appeared to be independent prognostic factors for survival (HR up to 1.5 and 2.2, respectively) and were also associated with neurophysiological markers of LMN damage, although only Cl^−^ demonstrated a correlation with disease progression rate. CK was linked with neurophysiological LMN signs and disability, but not with survival. CRP correlated with clinical LMN signs, but no other associations were found (Fig. 1).

Besides its use as a surrogate index of renal function, serum creatinine is also known to reflect muscle mass. In ALS, serum creatinine appears to decrease up to 2 years before diagnosis [18], with baseline values being consistently positively associated with progression and survival [4, 6, 19]. We confirmed that serum creatinine is a reliable marker of disease severity and mortality in ALS, with a 14% reduction in risk of death observed for each 0.1 mg/dL increase at diagnosis. Furthermore, baseline levels correlated with degree of LMN loss and disability. Several studies further demonstrated that longitudinal creatinine decrease mirrors muscle loss [6, 20] and highly correlates with disease progression and risk of mortality, potentially with a lower between-patient variability than ALSFRS-R scores [5], encouraging the use of creatinine as an additional outcome measure in clinical trials. In this view, the use of serum creatinine for routine estimation of renal function may be flawed by the dynamics of muscle loss. Alternative serum factors, such as cystatin C, may thus be helpful to better estimate glomerular filtration rate in ALS patients with suspected kidney disease [21].

Markers of nutrition and inflammation have also been linked with survival in ALS. Albumin appeared as the most consistent prognostic marker across several studies [6, 22, 23]. In our study, we found that albumin and CRP correlated with clinical LMN features, but only lower total protein levels and albumin were independently associated with worse survival in ALS patients. Total protein levels and albumin were initially used as separate variables, possibly accounting for the association observed only for total protein but not albumin. Indeed, after exclusion of total protein levels from the survival analysis, albumin was retained as an independent prognostic factor, consistent with the literature [4, 6, 22], suggesting that it may be a reasonable outcome measure in ALS clinical trials, as recently suggested in the Co-ALS trial [24–27]. Instead, despite some evidence for the presence of a systemic low-grade inflammation in ALS [7, 28, 29], we did not find an association between CRP and survival, similar to other large cohort studies [22, 30, 31]. Although we failed to observe any direct correlation between total protein and albumin with CRP, current evidence suggests that the former two analytes may be at least partially influenced by inflammatory processes. Therefore, it is possible that, besides their natural role in reflecting nutritional status, their significant association with ALS phenotype and survival may be due to their link with inflammation.

We showed that higher AST and lower Cl^−^ levels are negative independent prognostic factors in ALS, also correlating with neurophysiological LMN features and, for chloride, with disease progression rate as well (Fig. 1). Furthermore, K^+^ showed significant correlations with neurophysiological LMN signs, as well as with disease progression rate. These results were surprising, as most studies analysing the role of blood parameters in ALS, with the exception of two [32, 33], did not report any significant association with either liver indices or electrolytes. In support of a potential biological role for these parameters, we found significant associations with disease features and/or disability also for ALT and K^+^. Correlations of CK with AST and ALT suggest that changes in these blood analytes might be in our patients representative of muscle damage, although some studies reported evidence of liver dysfunction in ALS patients and animal models [34, 35], which deserves further investigation. Whereas AST and ALT may be functionally linked to muscle damage, identifying a biological explanation for the observed associations of electrolytes such as Cl^−^ and K^+^ with ALS is much trickier. Nonetheless, a recent study showed that serum Cl^−^, due to its relationship with acid–base balance, may represent an indirect biomarker of respiratory function in ALS patients and its levels are associated with survival and time to non-invasive ventilation [36].

Elevated CK levels may occur in 40–50% of ALS patients at diagnosis, being higher in males and in spinal-onset cases [37, 38], with similar figures observed in our cohort. Based on longitudinal data, chances of detecting abnormal CK levels are higher in the early stages of the disease [39] and decrease thereafter [40]. Notably, CK correlated more strongly with neurophysiological rather than clinical LMN scores, suggesting that CK better reflects the spatial extent of LMN loss and accompanying muscle damage, as shown by the cumulative active and chronic denervation across body regions, rather than the clinical severity of the disease itself. Indeed, studies assessing scores of either cumulative active denervation [41, 42] or spatial distribution (but not severity) of LMN burden [37] found a significant association with CK, while our clinical LMN score accounts for both distribution and severity of LMN damage [43]. We failed to detect a survival association with CK in our ALS cohort, in coherence with findings from other large ALS cohorts [4, 6, 37]. Based on these findings, CK may be useful in the early stages of the disease as a marker of widespread involvement but not for prognostic stratification and follow-up of ALS patients.

Uric acid and markers of lipid profile have also been associated with survival in ALS, though with only partial reproducibility across studies. Indeed, although associations at univariate analysis can be frequently found, as in our study, after adjustment for known prognostic factors, only few studies were still able to demonstrate an independent prognostic value [44–47]. Our results indicate that after adjustment for other blood factors, neither urate nor lipid biomarkers are significantly associated with risk of death in ALS. Nonetheless, we found a negative correlation between uric acid and UMN burden score, suggesting a possible role of uric acid as a marker of central neurodegeneration. Supporting this hypothesis, one study reported that lower urate levels predicted cognitive impairment in ALS patients [48].

This study has some limitations. Rather than a population-based cohort, we analyzed data from a referral center, a setting which is known to be affected by some biases, such as a longer overall survival and diagnostic delay. Nonetheless, our cohort presents many similarities with other Italian ALS cohorts [49, 50], supporting the validity and potential reproducibility of our data. Considering the high number of blood analytes tested in this study, correlation and survival analyses were filtered using correction for multiple testing and a stepwise approach for multivariate analysis to avoid simultaneous evaluation of all blood factors, which may compromise fitness and validity of the Cox model. Furthermore, our results were significant at the group level, but information about their usefulness on the individual patients is uncertain. We acknowledge that spurious associations may still be found, such as the very small risk protection conferred by higher LDH, whose biological significance is not clear. Nonetheless, we reported data based on the combination with different disease features and outcomes rather than on single significant *p*-values, looking for consistency and reproducibility of the associations found. Indeed, the AIC of the models including or excluding LDH was very similar. The combination of both known and new findings lends support to the validity of our results, although further studies are needed for replication and validation.

In conclusion, we confirm that valuable repurposing of easily quantifiable and inexpensive blood biomarkers is possible in ALS, offering novel perspectives on the disease both in research and clinical practice. Creatinine is a strong biochemical marker of the disease, correlating with LMN features, disability and survival. Biomarkers of nutritional status and inflammation, such as total protein, albumin and CRP, correlate with burden of LMN loss, although protein status alone may provide independent prognostic information. AST and chloride, which may reflect ongoing muscle damage and loss, may be new promising biomarkers to apply for stratifying patients and predicting mortality risk, but these new findings need validation in future studies.

### Supplementary Information

Below is the link to the electronic supplementary material.

Supplementary file1 (DOCX 139 KB)

## References

[1] Feldman EL (2022). Amyotrophic lateral sclerosis. Lancet.

[2] Verde F, Otto M, Silani V (2021). Neurofilament light chain as biomarker for amyotrophic lateral sclerosis and frontotemporal dementia. Front Neurosci.

[3] Verde F (2019). Neurofilament light chain in serum for the diagnosis of amyotrophic lateral sclerosis. J Neurol Neurosurg Psychiatry.

[4] Hertel N (2022). Analysis of routine blood parameters in patients with amyotrophic lateral sclerosis and evaluation of a possible correlation with disease progression-a multicenter study. Front Neurol.

[5] van Eijk RPA (2018). Monitoring disease progression with plasma creatinine in amyotrophic lateral sclerosis clinical trials. J Neurol Neurosurg Psychiatry.

[6] Chio A (2014). Amyotrophic lateral sclerosis outcome measures and the role of albumin and creatinine: a population-based study. JAMA Neurol.

[7] Lunetta C (2017). Serum C-reactive protein as a prognostic biomarker in amyotrophic lateral sclerosis. JAMA Neurol.

[8] Brooks BR (2000). El Escorial revisited: revised criteria for the diagnosis of amyotrophic lateral sclerosis. Amyotroph Lateral Scler Other Motor Neuron Disord.

[9] Woo JH (2014). Linear associations between clinically assessed upper motor neuron disease and diffusion tensor imaging metrics in amyotrophic lateral sclerosis. PLoS ONE.

[10] Devine MS (2016). Targeted assessment of lower motor neuron burden is associated with survival in amyotrophic lateral sclerosis. Amyotroph Lateral Scler Frontotemporal Degener.

[11] Maranzano A (2022). Upper motor neuron dysfunction is associated with the presence of behavioural impairment in patients with amyotrophic lateral sclerosis. Eur J Neurol.

[12] Colombo E (2022). Correlation between clinical phenotype and electromyographic parameters in amyotrophic lateral sclerosis. J Neurol.

[13] Cedarbaum JM (1999). The ALSFRS-R: a revised ALS functional rating scale that incorporates assessments of respiratory function. BDNF ALS Study Group (Phase III). J Neurol Sci.

[14] Ratti A (2022). Genetic and epigenetic disease modifiers in an Italian C9orf72 family expressing ALS, FTD or PD clinical phenotypes. Amyotroph Lateral Scler Frontotemporal Degener.

[15] Palmqvist S (2021). Prediction of future Alzheimer's disease dementia using plasma phospho-tau combined with other accessible measures. Nat Med.

[16] Burnham KP, Anderson DR (2016). Multimodel Inference. Sociol Methods Res.

[17] Slinker BK, Glantz SA (1985). Multiple regression for physiological data analysis: the problem of multicollinearity. Am J Physiol.

[18] Cui C (2020). Creatinine and C-reactive protein in amyotrophic lateral sclerosis, multiple sclerosis and Parkinson's disease. Brain Commun.

[19] Kuffner R (2015). Crowdsourced analysis of clinical trial data to predict amyotrophic lateral sclerosis progression. Nat Biotechnol.

[20] Holdom CJ (2021). Venous creatinine as a biomarker for loss of fat-free mass and disease progression in patients with amyotrophic lateral sclerosis. Eur J Neurol.

[21] Tetsuka S (2013). Utility of cystatin C for renal function in amyotrophic lateral sclerosis. Acta Neurol Scand.

[22] Sun J (2020). Blood biomarkers and prognosis of amyotrophic lateral sclerosis. Eur J Neurol.

[23] Chen X (2019). Clinical disease stage related changes of serological factors in amyotrophic lateral sclerosis. Amyotroph Lateral Scler Frontotemporal Degener.

[24] Mandrioli J (2019). Proteostasis and ALS: protocol for a phase II, randomised, double-blind, placebo-controlled, multicentre clinical trial for colchicine in ALS (Co-ALS). BMJ Open.

[25] Warnes TW (1987). A controlled trial of colchicine in primary biliary cirrhosis. Trial design and preliminary report. J Hepatol.

[26] Mirian A, Korngut L (2018). The utility of the laboratory work up at the time of diagnosis of amyotrophic lateral sclerosis. J Neuromuscul Dis.

[27] Bond L (2020). Associations of patient mood, modulators of quality of life, and pharmaceuticals with amyotrophic lateral sclerosis survival duration. Behav Sci (Basel).

[28] Beers DR (2020). Elevated acute phase proteins reflect peripheral inflammation and disease severity in patients with amyotrophic lateral sclerosis. Sci Rep.

[29] Kharel S (2022). C-reactive protein levels in patients with amyotrophic lateral sclerosis: a systematic review. Brain Behav.

[30] Nagel G (2017). Adipokines, C-reactive protein and amyotrophic lateral sclerosis—results from a population-based ALS registry in Germany. Sci Rep.

[31] De Schaepdryver M (2020). Neurofilament light chain and C reactive protein explored as predictors of survival in amyotrophic lateral sclerosis. J Neurol Neurosurg Psychiatry.

[32] Lunetta C (2015). Amyotrophic lateral sclerosis survival score (ALS-SS): a simple scoring system for early prediction of patient survival. Amyotroph Lateral Scler Frontotemporal Degener.

[33] Qureshi M (2008). Medications and laboratory parameters as prognostic factors in amyotrophic lateral sclerosis. Amyotroph Lateral Scler.

[34] Nakano Y, Hirayama K, Terao K (1987). Hepatic ultrastructural changes and liver dysfunction in amyotrophic lateral sclerosis. Arch Neurol.

[35] Lee SH, Yang EJ (2018). Relationship between liver pathology and disease progression in a murine model of amyotrophic lateral sclerosis. Neurodegener Dis.

[36] Manera U (2023). Serum chloride as a respiratory failure marker in amyotrophic lateral sclerosis. Front Aging Neurosci.

[37] Gao J (2022). Creatine kinase and prognosis in amyotrophic lateral sclerosis: a literature review and multi-centre cohort analysis. J Neurol.

[38] Rafiq MK (2016). Creatine kinase enzyme level correlates positively with serum creatinine and lean body mass, and is a prognostic factor for survival in amyotrophic lateral sclerosis. Eur J Neurol.

[39] Ito D (2019). Elevated serum creatine kinase in the early stage of sporadic amyotrophic lateral sclerosis. J Neurol.

[40] Chen XP (2021). Creatine kinase in the diagnosis and prognostic prediction of amyotrophic lateral sclerosis: a retrospective case-control study. Neural Regen Res.

[41] Tai H (2017). Correlation of creatine kinase levels with clinical features and survival in amyotrophic lateral sclerosis. Front Neurol.

[42] Tai H (2018). Creatine kinase level and its relationship with quantitative electromyographic characteristics in amyotrophic lateral sclerosis. Clin Neurophysiol.

[43] Colombo E (2022). Motor, cognitive and behavioural profiles of *C9orf72* expansion-related amyotrophic lateral sclerosis. J Neurol.

[44] Ingre C (2020). Lipids, apolipoproteins, and prognosis of amyotrophic lateral sclerosis. Neurology.

[45] Paganoni S (2018). Urate levels predict survival in amyotrophic lateral sclerosis: analysis of the expanded pooled resource open-access ALS clinical trials database. Muscle Nerve.

[46] Thompson AG (2022). Multicentre appraisal of amyotrophic lateral sclerosis biofluid biomarkers shows primacy of blood neurofilament light chain. Brain Commun.

[47] Yazdani S (2019). Peripheral immune biomarkers and neurodegenerative diseases: a prospective cohort study with 20 years of follow-up. Ann Neurol.

[48] Tang J (2021). Plasma uric acid helps predict cognitive impairment in patients with amyotrophic lateral sclerosis. Front Neurol.

[49] Chio A (2011). Phenotypic heterogeneity of amyotrophic lateral sclerosis: a population based study. J Neurol Neurosurg Psychiatry.

[50] Falzone YM (2022). Integrated evaluation of a panel of neurochemical biomarkers to optimize diagnosis and prognosis in amyotrophic lateral sclerosis. Eur J Neurol.

