# Supplementary material for: The value of routine blood work-up in clinical stratification and prognosis of patients with amyotrophic lateral sclerosis
Source: J Neurol. 2023 Oct 6;271(2):794–803. doi: 10.1007/s00415-023-12015-3 (PMC10827966; doi:10.1007/s00415-023-12015-3)
Supplement: Supplementary file 1 — Supplementary file1 (DOCX 139 KB) [file 415_2023_12015_MOESM1_ESM.docx]

**Supplementary Table S1. Frequency of abnormalities and differences according to site of onset**

|  | **N** | **Normal range** | **%Ab** | **Spinal,**  **Median (r)** | **Bulbar,**  **Median (r)** | **adj *p*-value** |
| --- | --- | --- | --- | --- | --- | --- |
| WBC (10^9/L) | 836 | 4.3-10.5 | 9.3% | 6.3 (2.9-15.4) | 6.1 (1.4-13.6) | ns |
| RBC (10^12/L) | 836 | 4.5-6.2 (M)  4.1-5.5 (F) | 26.7% | 4.64 (2.91-6.53) | 4.49 (3.4-6.97) | ns |
| Hb (g/dL) M | 836 | 13.8-18 (M)  11.9-16 (F) | 20.6% | 14.15 (8.4-19) | 13.55 (9.5-17.5) | **0.04** |
| PLT (10^9/L) | 836 | 140-450 | 5.9% | 215 (55-480) | 212.5 (15-575) | ns |
| PMN (10^9/L) | 836 | 1.8-8.1 | 4.1% | 3.7 (1.1-13.1) | 3.4 (0.9-9.4) | ns |
| Ly (10^9/L) | 836 | 0.9-5.4 | 1.8% | 1.8 (0.4-5) | 1.7 (0.4-4.4) | ns |
| Mo (10^9/L) | 836 | 0.1-1.5 | 0% | 0.5 (0.2-1.3) | 0.5 (0.1-1) | **0.01** |
| B12 (ng/L) | 731 | 211-911 | 14.9% | 443 (100-4000) | 431 (85-4000) | ns |
| ESR (mm/h) | 828 | <15 | 28.4% | 9 (2-120) | 10.5 (2-67) | ns |
| AST (U/L) | 836 | <40 | 7.8% | 23 (4-112) | 20.5 (9-97) | **0.02** |
| ALT (U/L) | 836 | <40 | 14.2% | 24 (4-127) | 18 (6-118) | **1.8e-06** |
| ALP (U/L) | 830 | 40-130 | 5.9% | 61 (24-547) | 63 (21-148) | ns |
| GGT (U/L) | 821 | <60 | 6.0% | 21 (5-802) | 17 (7-166) | ns |
| LDH (U/L) | 831 | <250 | 59.0% | 293 (72-8862) | 291 (116-545) | ns |
| CK (U/L) | 835 | <190 | 44.8% | 201 (16-3768) | 109.5 (26-891) | **3.4e-08** |
| CHE (U/L) | 798 | 4000-14000 | 1.8% | 7765.5 (1820-18083) | 7604 (2752-12921) | ns |
| Urea (mg/dL) | 821 | 10-50 | 8.5% | 36 (10-83) | 37 (8-76) | ns |
| Creatinine (mg/dL) | 832 | 0.4-1.4 | 1.8%* | 0.7 (0.1-1.4) | 0.8 (0.4-1.4) | **3.6e-07** |
| Urate (mg/dL) | 823 | 2.0-7.0 | 5.5% | 4.9 (1.2-10.3) | 4.5 (1.5-10.6) | ns |
| Bilirubine (mg/dL) | 819 | 0.1-1.15 | 9.2% | 0.62 (0.18-3.56) | 0.63 (0.15-2.87) | ns |
| Tot proteins (g/dL) | 834 | 6.5-8.5 | 35.1%* | 6.7 (5.2-9.4) | 6.75 (5.5-8.4) | ns |
| Albumin (g/dL) | 833 | 3.5-5.5 | 6.5% | 4.03 (2.7-5.2) | 4.03 (3.1-4.97) | ns |
| Tot cholesterol (mg/dL) | 821 | <200 | 51.2% | 202 (109-397) | 203 (110-331) | ns |
| HDL (mg/dL) | 824 | >40 | 13.2% | 53 (25-145) | 57 (29-130) | ns |
| LDL (mg/dL) | 824 | <115 | 62.0% | 127 (22-277.2) | 125 (32-240) | ns |
| TG (mg/dL) | 825 | <150 | 18.9% | 106 (29-625) | 92 (35-749) | ns |
| Na^+^ (mmol/L) | 836 | 136-147 | 1.4% | 142 (126-149) | 142 (136-148) | ns |
| K^+^ (mmol/L) | 834 | 3.5-5.4 | 2.4% | 4.2 (3-5.9) | 4.3 (3.4-5.3) | ns |
| Cl^-^ (mmol/L) | 832 | 98-112 | 3.2% | 104 (88-206) | 104 (97-111) | ns |
| Ca^2+^ (mg/dL) | 800 | 8.1-10.4 | 1.0% | 9.3 (8-11.3) | 9.3 (8.2-10.9) | ns |
| CRP (mg/dL) | 576 | <1.00 | 4.5% | 0.1 (0-6.8) | 0.1 (0-2) | ns |
| Glucose (mg/dL) | 830 | 70-100 | 25.5% | 92 (35-354) | 89 (70-172) | ns |
| TSH (mU/L) | 826 | 0.27-4.5 | 4.4% | 1.63 (0.02-11.3) | 1.47 (0.03-10.6) | ns |
| Data are presented in number (percentage) or median (range). *p*-values were adjusted for multiple testing (66 comparisons). * Intended as low levels. %Ab: % abnormal. Significant sex differences were found for: WBC (0.009), RBC (4.6e-20), Hb (3.7e-36), Plt (5.1e-8), PMN (0.003), Mo (1e-06), ESR (4.2e-7), AST (0.003), ALT (2e-6), ALP (0.005), GGT (6.6e-11), LDH (0.027), CPK (1.1e-11), urea (0.001), creatinine (3.3e-31), uric acid (1.2e-27), bilirubin (1.8e-14), albumin (0.013), CHO (0.001), HDL (2.97e-21), TG (8.4e-7), Na (0.02), K (0.02), Cl (0.0002), Ca (0.04), glucose (0.006). | | | | | | |

**Supplementary Table S2. Expanded correlations of blood tests with UMN and LMN signs**

|  | **UMN score**  **(n=776)** | | **MRC score**  **(n=640)** | | **LMN score**  **(n=776)** | | **AD score**  **(n=699)** | | **CD score**  **(n=699)** | |
| --- | --- | --- | --- | --- | --- | --- | --- | --- | --- | --- |
|  | **rho** | ***p*** | **rho** | ***p*** | **rho** | ***p*** | **rho** | ***p*** | **rho** | ***p*** |
| WBC | -0.01 | ns | -0.04 | ns | 0.06 | ns | 0.05 | ns | 0.05 | ns |
| RBC | -0.11 | ns | 0.03 | ns | -0.05 | ns | 0.09 | ns | 0.09 | ns |
| Hb | -0.12 | ns | 0.06 | ns | -0.06 | ns | 0.11 | ns | 0.09 | ns |
| PLT | 0 | ns | -0.09 | ns | 0.06 | ns | 0.05 | ns | 0.05 | ns |
| PMN | -0.01 | ns | -0.06 | ns | 0.08 | ns | 0.09 | ns | 0.07 | ns |
| Ly | 0.02 | ns | 0 | ns | -0.03 | ns | -0.05 | ns | -0.04 | ns |
| Mo | -0.08 | ns | -0.06 | ns | 0.09 | ns | 0.05 | ns | 0.06 | ns |
| B12 | 0.08 | ns | -0.1 | ns | 0.11 | ns | 0.08 | ns | 0.07 | ns |
| VES | 0.05 | ns | -0.12 | ns | 0.11 | ns | 0.06 | ns | 0.05 | ns |
| AST | **-0.18** | **1.4e-04** | -0.11 | ns | 0.07 | ns | **0.24** | **1.6e-08** | **0.19** | **5.1e-05** |
| ALT | -0.12 | ns | **-0.21** | **1.2e-05** | 0.11 | ns | **0.27** | **9.6e-11** | **0.2** | **2.1e-05** |
| ALP | 0.03 | ns | -0.04 | ns | 0.02 | ns | -0.05 | ns | -0.05 | ns |
| GGT | -0.08 | ns | -0.08 | ns | 0.07 | ns | 0.12 | ns | 0.09 | ns |
| LDH | -0.06 | ns | -0.07 | ns | 0.02 | ns | 0 | ns | 0.05 | ns |
| CK | **-0.31** | **8.7e-17** | **-0.15** | **2.0e-02** | 0.09 | ns | **0.35** | **7.1e-19** | **0.37** | **2.8e-21** |
| CHE | -0.09 | ns | -0.04 | ns | -0.06 | ns | 0.1 | ns | 0.03 | ns |
| urea | -0.06 | ns | -0.05 | ns | 0 | ns | -0.02 | ns | 0.01 | ns |
| creatinine | 0.05 | ns | **0.41** | **2.0e-25** | **-0.38** | **2.6e-25** | **-0.18** | **1.8e-04** | **-0.24** | **1.3e-08** |
| Uric acid | **-0.16** | **1.5e-03** | 0.05 | ns | -0.07 | ns | 0.09 | ns | 0.06 | ns |
| Bilirubin | -0.01 | ns | 0.01 | ns | 0.04 | ns | 0.08 | ns | 0.09 | ns |
| Tot. proteins | -0.03 | ns | 0.04 | ns | -0.09 | ns | -0.01 | ns | 0.01 | ns |
| Albumin | -0.02 | ns | 0.14 | ns | **-0.2** | **5.9e-04** | -0.02 | ns | -0.03 | ns |
| CHO | 0.01 | ns | -0.02 | ns | -0.02 | ns | 0.01 | ns | 0 | ns |
| HDL | 0.02 | ns | 0.1 | ns | -0.06 | ns | -0.09 | ns | -0.05 | ns |
| LDL | 0 | ns | -0.04 | ns | -0.01 | ns | 0.02 | ns | 0.02 | ns |
| TG | -0.08 | ns | -0.11 | ns | 0.04 | ns | 0.09 | ns | 0.06 | ns |
| Na^+^ | -0.03 | ns | 0.04 | ns | -0.04 | ns | -0.08 | ns | -0.05 | ns |
| K^+^ | -0.05 | ns | 0.13 | ns | -0.12 | ns | **-0.17** | **1.2e-03** | -0.07 | ns |
| Cl^-^ | 0.05 | ns | 0.06 | ns | **-0.15** | **7.1e-03** | **-0.18** | **4.6e-04** | **-0.19** | **1.0e-04** |
| Ca^2+^ | 0.04 | ns | -0.07 | ns | 0.01 | ns | 0.07 | ns | 0.04 | ns |
| CRP | -0.01 | ns | **-0.21** | **1.1e-03** | **0.17** | **2.0e-02** | 0.1 | ns | 0.12 | ns |
| glucose | **-0.15** | **4.5e-03** | 0.04 | ns | -0.04 | ns | 0.13 | ns | 0.07 | ns |
| TSH | 0 | ns | 0.01 | ns | -0.02 | ns | 0.01 | ns | -0.06 | ns |
| Spearman’s correlation. p-values were Bonferroni-adjusted for multiple testing (165 comparisons). | | | | | | | | | | |

**Supplementary Table S3. Expanded correlations of blood tests with disability and progression rate**

|  | **ALSFRS-R tot (n=538)** | | **ALSFRS-R M (n=450)** | | **ALSFRS-R B (n=450)** | | **ALSFRS-R R (n=450)** | | **Δ ALSFRS-R (n=538)** | |
| --- | --- | --- | --- | --- | --- | --- | --- | --- | --- | --- |
|  | **rho** | ***p*** | **rho** | ***p*** | **rho** | ***p*** | **rho** | ***p*** | **rho** | ***p*** |
| WBC | -0.06 | ns | -0.11 | ns | 0.15 | ns | -0.07 | ns | -0.04 | ns |
| RBC | 0.09 | ns | 0.01 | ns | 0.17 | ns | 0 | ns | 0 | ns |
| Hb | 0.14 | ns | 0.03 | ns | **0.2** | **2.0e-03** | 0 | ns | -0.02 | ns |
| PLT | -0.04 | ns | -0.06 | ns | 0.07 | ns | 0.03 | ns | 0.02 | ns |
| PMN | -0.09 | ns | -0.15 | ns | 0.11 | ns | -0.09 | ns | -0.01 | ns |
| Ly | 0.05 | ns | 0.03 | ns | 0.1 | ns | 0.04 | ns | -0.06 | ns |
| Mo | -0.03 | ns | -0.09 | ns | 0.1 | ns | -0.09 | ns | -0.01 | ns |
| B12 | -0.12 | ns | -0.13 | ns | 0.01 | ns | 0.03 | ns | 0.07 | ns |
| VES | -0.15 | ns | -0.14 | ns | 0.02 | ns | -0.03 | ns | 0.08 | ns |
| AST | 0.05 | ns | -0.05 | ns | 0.15 | ns | 0.01 | ns | 0 | ns |
| ALT | -0.01 | ns | -0.13 | ns | **0.24** | **7.1e-05** | 0.03 | ns | 0.02 | ns |
| ALP | -0.14 | ns | -0.12 | ns | -0.04 | ns | -0.02 | ns | 0.02 | ns |
| GGT | -0.04 | ns | -0.08 | ns | 0.15 | ns | -0.05 | ns | 0.01 | ns |
| LDH | 0.08 | ns | 0.06 | ns | 0.07 | ns | -0.04 | ns | -0.09 | ns |
| CK | **0.18** | **7.3e-03** | 0 | ns | **0.33** | **2.3e-10** | 0.02 | ns | -0.09 | ns |
| CHE | 0.08 | ns | 0.02 | ns | 0.13 | ns | 0.02 | ns | -0.01 | ns |
| urea | 0.01 | ns | -0.08 | ns | 0.05 | ns | 0.03 | ns | 0.03 | ns |
| creatinine | **0.33** | **1.5e-12** | **0.34** | **4.0e-11** | -0.01 | ns | 0.12 | ns | -0.06 | ns |
| Uric acid | 0.09 | ns | -0.01 | ns | **0.21** | **1.2e-03** | 0.07 | ns | -0.05 | ns |
| Bilirubin | 0 | ns | 0 | ns | 0.04 | ns | -0.03 | ns | 0.05 | ns |
| Tot. proteins | 0.06 | ns | 0 | ns | 0.04 | ns | 0.09 | ns | -0.02 | ns |
| Albumin | 0.15 | ns | 0.08 | ns | 0.05 | ns | 0.11 | ns | -0.07 | ns |
| CHO | 0.01 | ns | -0.03 | ns | 0.09 | ns | 0.09 | ns | -0.03 | ns |
| HDL | 0.02 | ns | 0.06 | ns | -0.14 | ns | -0.01 | ns | -0.04 | ns |
| LDL | 0 | ns | -0.07 | ns | 0.13 | ns | 0.1 | ns | -0.01 | ns |
| TG | -0.04 | ns | -0.09 | ns | 0.14 | ns | 0 | ns | 0 | ns |
| Na^+^ | 0.03 | ns | 0.09 | ns | -0.03 | ns | -0.07 | ns | -0.08 | ns |
| K^+^ | **0.17** | **1.4e-02** | 0.14 | ns | 0.05 | ns | 0.07 | ns | **-0.19** | **1.1e-03** |
| Cl^-^ | 0.15 | ns | 0.16 | ns | 0.06 | ns | 0.12 | ns | **-0.16** | **3.8e-02** |
| Ca^2+^ | -0.15 | ns | **-0.22** | **4.3e-04** | -0.02 | ns | 0.05 | ns | 0.15 | ns |
| CRP | -0.14 | ns | -0.17 | ns | 0.1 | ns | -0.12 | ns | 0.02 | ns |
| glucose | 0.07 | ns | 0 | ns | 0.11 | ns | -0.04 | ns | 0 | ns |
| TSH | 0 | ns | -0.05 | ns | 0.03 | ns | -0.08 | ns | 0.04 | ns |
| Spearman’s correlation. p-values were Bonferroni-adjusted for multiple testing (165 comparisons). | | | | | | | | | | |

**Supplementary table S4. Cox regression analysis of single blood analytes**

| **Cox survival regression analysis of blood parameters in ALS.** | | | | | | | | | |
| --- | --- | --- | --- | --- | --- | --- | --- | --- | --- |
| **Factor** | **HR (95% CI)** | ***p*** | **Factor** | | **HR (95% CI)** | ***p*** | **Factor** | **HR (95% CI)** | ***p*** |
| WBC | 0.96 (0.91-1.02) | 0.17 | ALP | 0.99 (0.99-1.003) | | 0.67 | CHO | 0.998 (0.996-1.001) | 0.19 |
| RBC | 0.85 (0.67-1.07) | 0.17 | GGT | 1.003 (1-1.005) | | **0.022** | HDL | 1.001 (0.99-1.007) | 0.79 |
| Hb | 0.96 (0.88-1.05) | 0.39 | LDH | 0.998 (0.997-0.999) | | **0.002** | LDL | 0.99 (0.995-1.001) | 0.15 |
| PLT | 0.99 (0.998-1.001) | 0.47 | CK | 1 (1-1.001) | | 0.48 | TG | 1.001 (0.99-1.002) | 0.48 |
| PMN | 0.96 (0.90-1.04) | 0.32 | CHE | 1 (1-1) | | 0.55 | Na^+^ | 0.97 (0.93-1.02) | 0.27 |
| Ly | 0.90 (0.75-1.08) | 0.23 | urea | 0.986 (0.974-0.998) | | **0.025** | K^+^ | 0.70 (0.53-0.92) | **0.01** |
| Mo | 0.79 (0.39-1.59) | 0.51 | creatinine | 0.86 (0.81-0.92) | | **<0.001** | Cl^-^ | 0.95 (0.92-0.99) | **0.005** |
| B12 | 1 (1-1) | 0.82 | Uric acid | 0.88 (0.81-0.95) | | **0.001** | Ca^2+^ | 1.25 (0.97-1.62) | 0.08 |
| ESR | 1.01 (0.99-1.02) | 0.09 | bilirubin | 1.03 (0.78-1.36) | | 0.83 | CRP | 1.02 (0.87-1.2) | 0.80 |
| AST | 1.012 (1.005-1.02) | **0.001** | Total proteins | 0.82 (0.69-0.98) | | **0.033** | Glucose | 1.004 (0.99-1.009) | 0.09 |
| ALT | 1.01 (1.004-1.016) | **0.002** | Albumin | 0.74 (0.58-0.95) | | **0.018** | TSH | 0.89 (0.81-0.97) | **0.01** |
| Hazard ratio and 95% confidence intervals of blood parameters were all adjusted for time to first evaluation, age of onset and presence of *C9orf72* repeat expansion. | | | | | | | | | |

**Supplementary table S5. Multivariate Cox regression analysis of blood analytes in ALS**

|  | **First phase multivariate analysis** | | **Last phase multivariate analysis** | |
| --- | --- | --- | --- | --- |
|  | **HR (95% CI)** | ***p*** | **HR (95% CI)** | ***p*** |
| **Age of onset** | 1.029 (1.017-1.041) | <0.001 | 1.028 (1.017-1.039) | **<0.001** |
| ***C9orf72* hre** | 1.835 (1.226-2.748) | 0.003 | 1.867 (1.252-2.782) | **0.002** |
| **Time to first evaluation** | 0.934 (0.924-0.944) | <0.001 | 0.934 (0.925-0.945) | **<0.001** |
| **AST** | 1.015 (0.998-1.033) | 0.081 | 1.017 (1.009-1.024) | **<0.001** |
| **ALT** | 1.001 (0.987-1.016) | 0.841 |  |  |
| **GGT** | 1.002 (0.999-1.005) | 0.175 |  |  |
| **LDH** | 0.998 (0.997-0.999) | 0.002 | 0.998 (0.997-0.999) | **0.001** |
| **Urea** | 1.001 (0.987-1.014) | 0.913 |  |  |
| **Creatinine*** | 0.886 (0.82-0.956) | 0.002 | 0.862 (0.81-0.918) | **<0.001** |
| **Urate** | 0.97 (0.88-1.069) | 0.542 |  |  |
| **Total protein levels** | 0.732 (0.586-0.915) | 0.006 | 0.699 (0.569-0.858) | **0.001** |
| **Albumin** | 1.002 (0.973-1.032) | 0.908 |  |  |
| **K^+^** | 0.779 (0.567-1.07) | 0.123 |  |  |
| **Cl^-^** | 0.955 (0.919-0.992) | 0.018 | 0.952 (0.918-0.988) | **0.009** |
| **TSH** | 0.918 (0.833-1.013) | 0.089 |  |  |
| Prognostic role of routine blood parameters in ALS, assessed as continuous variables. A backward conditional Cox regression analysis was conducted in the study cohort. *per 0.1 mg/dL unit change. *hre*: hexanucleotide expansion repeat. | | | | |

**Supplementary Table S6. Inter-blood parameter correlations**

|  | **WBC** | | **RBC** | | **Hb** | | **PLT** | | **PMN** | | **Ly** | | **Mo** | | **B12** | | **ESR** | | **AST** | | **ALT** | |
| --- | --- | --- | --- | --- | --- | --- | --- | --- | --- | --- | --- | --- | --- | --- | --- | --- | --- | --- | --- | --- | --- | --- |
|  | **rho** | ***p*** | **rho** | ***p*** | **rho** | ***p*** | **rho** | ***p*** | **rho** | ***p*** | **rho** | ***p*** | **rho** | ***p*** | **rho** | ***p*** | **rho** | ***p*** | **rho** | ***p*** | **rho** | ***p*** |
| RBC | **0.22** | **1.0e-07** | 1 |  | **0.74** | **4.7e-144** | 0.02 | ns | **0.26** | **6.5e-11** | 0.03 | ns | 0.12 | ns | 0.08 | ns | **-0.18** | **2.3e-04** | 0.13 | ns | **0.22** | **5.6e-08** |
| Hb | **0.23** | **3.0e-08** | **0.74** | **4.7e-144** | 1 |  | 0 | ns | **0.24** | **3.7e-09** | 0.06 | ns | **0.18** | **3.0e-04** | 0.07 | ns | **-0.28** | **7.1e-13** | **0.24** | **2.9e-09** | **0.32** | **4.6e-18** |
| PLT | **0.28** | **1.3e-13** | 0.02 | ns | 0 | ns | 1 |  | **0.25** | **1.0e-10** | 0.13 | ns | 0.11 | ns | 0.12 | ns | **0.17** | **1.6e-03** | 0.03 | ns | 0.07 | ns |
| PMN | **0.89** | **6.5e-279** | **0.26** | **6.5e-11** | **0.24** | **3.7e-09** | **0.25** | **1.0e-10** | 1 |  | 0.03 | ns | **0.41** | **1.4e-31** | 0.12 | ns | **0.19** | **4.1e-05** | 0.01 | ns | 0.1 | ns |
| Ly | **0.42** | **3.8e-33** | 0.03 | ns | 0.06 | ns | 0.13 | ns | 0.03 | ns | 1 |  | **0.29** | **4.1e-15** | 0.01 | ns | -0.04 | ns | -0.07 | ns | -0.02 | ns |
| Mo | **0.57** | **7.0e-70** | 0.12 | ns | **0.18** | **3.0e-04** | 0.11 | ns | **0.41** | **1.4e-31** | **0.29** | **4.1e-15** | 1 |  | 0.03 | ns | 0.04 | ns | 0.02 | ns | 0.07 | ns |
| B12 | 0.1 | ns | 0.08 | ns | 0.07 | ns | 0.12 | ns | 0.12 | ns | 0.01 | ns | 0.03 | ns | 1 |  | 0.05 | ns | **0.23** | **2.1e-07** | **0.27** | **1.4e-10** |
| ESR | **0.16** | **3.6e-03** | **-0.18** | **2.3e-04** | **-0.28** | **7.1e-13** | **0.17** | **1.6e-03** | **0.19** | **4.1e-05** | -0.04 | ns | 0.04 | ns | 0.05 | ns | 1 |  | -0.05 | ns | -0.03 | ns |
| AST | -0.01 | ns | 0.13 | ns | **0.24** | **2.9e-09** | 0.03 | ns | 0.01 | ns | -0.07 | ns | 0.02 | ns | **0.23** | **2.1e-07** | -0.05 | ns | 1 |  | **0.78** | **1.4e-166** |
| ALT | 0.09 | ns | **0.22** | **5.6e-08** | **0.32** | **4.6e-18** | 0.07 | ns | 0.1 | ns | -0.02 | ns | 0.07 | ns | **0.27** | **1.4e-10** | -0.03 | ns | **0.78** | **1.4e-166** | 1 |  |
| ALP | **0.22** | **2.6e-07** | 0.04 | ns | 0.03 | ns | **0.15** | **2.1e-02** | **0.26** | **1.4e-11** | -0.02 | ns | 0.06 | ns | 0.09 | ns | **0.26** | **9.0e-11** | 0.04 | ns | 0.09 | ns |
| GGT | **0.19** | **4.8e-05** | **0.17** | **9.1e-04** | **0.28** | **9.5e-14** | 0.06 | ns | **0.16** | **5.3e-03** | 0.11 | ns | 0.13 | ns | 0.13 | ns | 0.04 | ns | **0.33** | **2.7e-19** | **0.49** | **8.4e-49** |
| LDH | 0.06 | ns | 0.01 | ns | 0.13 | ns | **0.15** | **2.6e-02** | 0.07 | ns | 0.02 | ns | -0.05 | ns | 0.14 | ns | 0.05 | ns | **0.4** | **6.5e-31** | **0.31** | **2.2e-16** |
| CK | 0.02 | ns | **0.22** | **8.6e-08** | **0.29** | **3.2e-14** | 0 | ns | 0.03 | ns | -0.03 | ns | 0.05 | ns | 0.02 | ns | **-0.15** | **2.3e-02** | **0.54** | **3.8e-62** | **0.42** | **2.7e-33** |
| CHE | **0.15** | **2.5e-02** | **0.25** | **5.4e-10** | **0.26** | **9.1e-11** | 0.14 | ns | **0.16** | **1.2e-02** | 0.1 | ns | -0.02 | ns | 0.07 | ns | 0.05 | ns | 0.08 | ns | **0.17** | **2.4e-03** |
| urea | 0.02 | ns | -0.03 | ns | 0 | ns | -0.07 | ns | 0.04 | ns | -0.02 | ns | 0.06 | ns | 0.06 | ns | 0.06 | ns | 0.1 | ns | 0.07 | ns |
| creatinine | 0 | ns | 0.09 | ns | **0.18** | **3.2e-04** | **-0.18** | **3.0e-04** | -0.04 | ns | 0.05 | ns | 0.08 | ns | -0.1 | ns | **-0.14** | **4.4e-02** | 0.04 | ns | -0.02 | ns |
| Uric acid | 0.13 | ns | **0.24** | **3.8e-09** | **0.28** | **5.1e-13** | -0.07 | ns | 0.12 | ns | 0.07 | ns | 0.14 | ns | -0.06 | ns | -0.02 | ns | 0.08 | ns | 0.13 | ns |
| Bilirubin | -0.03 | ns | **0.15** | **1.4e-02** | **0.19** | **2.5e-05** | -0.13 | ns | -0.03 | ns | 0 | ns | 0.06 | ns | 0.03 | ns | **-0.16** | **3.6e-03** | **0.18** | **3.3e-04** | **0.17** | **1.1e-03** |
| Tot. prot. | **0.21** | **1.9e-06** | **0.32** | **9.0e-19** | **0.3** | **4.6e-16** | **0.23** | **3.0e-08** | **0.3** | **1.3e-15** | -0.09 | ns | 0 | ns | **0.18** | **1.7e-03** | **0.19** | **2.6e-05** | **0.18** | **1.3e-04** | **0.16** | **6.6e-03** |
| Albumin | 0.11 | ns | **0.39** | **4.2E-28** | **0.40** | **1.9E-29** | 0.11 | ns | **0.19** | **5.7E-05** | -0.07 | ns | -0.07 | ns | 0.12 | ns | **-0.14** | **0.04** | **0.16** | **0.01** | **0.15** | **0.01** |
| CHO | 0.09 | ns | **0.15** | **2.9e-02** | **0.18** | **1.2e-04** | **0.2** | **3.5e-06** | 0.07 | ns | 0.13 | ns | -0.05 | ns | 0.1 | ns | 0.11 | ns | 0.1 | ns | 0.13 | ns |
| HDL | -0.11 | ns | **-0.16** | **2.7e-03** | **-0.15** | **3.2e-02** | **0.16** | **8.1e-03** | -0.06 | ns | -0.13 | ns | -0.09 | ns | 0.13 | ns | -0.03 | ns | 0.08 | ns | -0.07 | ns |
| LDL | 0.08 | ns | **0.19** | **2.0e-05** | **0.2** | **1.4e-05** | 0.13 | ns | 0.06 | ns | 0.13 | ns | -0.02 | ns | 0.04 | ns | 0.1 | ns | 0.03 | ns | 0.08 | ns |
| TG | **0.19** | **8.1e-05** | **0.2** | **4.2e-06** | **0.26** | **4.6e-11** | 0.03 | ns | **0.16** | **5.0e-03** | **0.16** | **3.0e-03** | 0.06 | ns | -0.04 | ns | 0.09 | ns | 0.08 | ns | **0.21** | **5.8e-07** |
| Na^+^ | -0.05 | ns | 0.01 | ns | -0.01 | ns | -0.09 | ns | -0.06 | ns | 0 | ns | -0.04 | ns | -0.08 | ns | -0.11 | ns | -0.06 | ns | -0.07 | ns |
| K^+^ | 0.06 | ns | 0.1 | ns | 0.1 | ns | 0.09 | ns | 0.08 | ns | -0.03 | ns | 0.1 | ns | 0.04 | ns | -0.01 | ns | 0.01 | ns | 0 | ns |
| Cl^-^ | **-0.15** | **8.1e-03** | **-0. 17** | **6.2e-04** | **-0.16** | **2.8e-03** | -0.11 | ns | **-0.2** | **8.2e-06** | 0.07 | ns | -0.14 | ns | -0.09 | ns | -0.09 | ns | -0.06 | ns | -0.06 | ns |
| Ca^2+^ | 0.14 | ns | **0.21** | **1.5e-06** | **0.15** | **3.0e-02** | **0.19** | **1.4e-04** | **0.19** | **9.3e-05** | -0.03 | ns | 0.03 | ns | 0.12 | ns | 0.04 | ns | 0.06 | ns | 0.08 | ns |
| CRP | **0.29** | **3.2e-09** | 0.07 | ns | 0.01 | ns | 0.09 | ns | **0.31** | **2.9e-11** | 0 | ns | **0.23** | **1.7e-05** | 0.05 | ns | **0.37** | **1.7e-17** | 0.02 | ns | 0.06 | ns |
| glucose | **0.17** | **4.4e-04** | **0.17** | **6.3e-04** | **0.19** | **5.2e-05** | 0.11 | ns | **0.25** | **3.0e-10** | -0.11 | ns | 0.08 | ns | -0.01 | ns | 0.09 | ns | 0.06 | ns | 0.09 | ns |
| TSH | 0.03 | ns | 0.03 | ns | 0.06 | ns | -0.03 | ns | 0.01 | ns | 0.08 | ns | 0.02 | ns | 0.01 | ns | -0.01 | ns | 0.09 | ns | 0.07 | ns |

**Supplementary Table S6 *(cont.)***

|  | **ALP** | | **GGT** | | **LDH** | | **CK** | | **CHE** | | **urea** | | **creatinine** | | **Uric acid** | | **bilirubin** | | **Tot. prot.** | | **Albumin** | |
| --- | --- | --- | --- | --- | --- | --- | --- | --- | --- | --- | --- | --- | --- | --- | --- | --- | --- | --- | --- | --- | --- | --- |
|  | **rho** | ***p*** | **rho** | ***p*** | **rho** | ***p*** | **rho** | ***p*** | **rho** | ***p*** | **rho** | ***p*** | **rho** | ***p*** | **rho** | ***p*** | **rho** | ***p*** | **rho** | ***p*** | **rho** | ***p*** |
| RBC | 0.04 | ns | **0.17** | **9.1e-04** | 0.01 | ns | **0.22** | **8.6e-08** | **0.25** | **5.4e-10** | -0.03 | ns | 0.09 | ns | **0.24** | **3.8e-09** | **0.15** | **1.4e-02** | **0.32** | **9.0e-19** | **0.39** | **4.2e-28** |
| Hb | 0.03 | ns | **0.28** | **9.5e-14** | 0.13 | ns | **0.29** | **3.2e-14** | **0.26** | **9.1e-11** | 0 | ns | **0.18** | **3.2e-04** | **0.28** | **5.1e-13** | **0.19** | **2.5e-05** | **0.3** | **4.6e-16** | **0.40** | **1.9e-29** |
| PLT | **0.15** | **2.1e-02** | 0.06 | ns | **0.15** | **2.6e-02** | 0 | ns | 0.14 | ns | -0.07 | ns | **-0.18** | **3.0e-04** | -0.07 | ns | -0.13 | ns | **0.23** | **3.0e-08** | 0.11 | ns |
| PMN | **0.26** | **1.4e-11** | **0.16** | **5.3e-03** | 0.07 | ns | 0.03 | ns | **0.16** | **1.2e-02** | 0.04 | ns | -0.04 | ns | 0.12 | ns | -0.03 | ns | **0.3** | **1.3e-15** | **0.19** | **5.7e-05** |
| Ly | -0.02 | ns | 0.11 | ns | 0.02 | ns | -0.03 | ns | 0.1 | ns | -0.02 | ns | 0.05 | ns | 0.07 | ns | 0 | ns | -0.09 | ns | -0.07 | ns |
| Mo | 0.06 | ns | 0.13 | ns | -0.05 | ns | 0.05 | ns | -0.02 | ns | 0.06 | ns | 0.08 | ns | 0.14 | ns | 0.06 | ns | 0 | ns | -0.07 | ns |
| B12 | 0.09 | ns | 0.13 | ns | 0.14 | ns | 0.02 | ns | 0.07 | ns | 0.06 | ns | -0.1 | ns | -0.06 | ns | 0.03 | ns | **0.18** | **1.7e-03** | 0.12 | ns |
| ESR | **0.26** | **9.0e-11** | 0.04 | ns | 0.05 | ns | **-0.15** | **2.3e-02** | 0.05 | ns | 0.06 | ns | **-0.14** | **4.4e-02** | -0.02 | ns | **-0.16** | **3.6e-03** | **0.19** | **2.6e-05** | **-0.14** | **3.9e-02** |
| AST | 0.04 | ns | **0.33** | **2.7e-19** | **0.4** | **6.5e-31** | **0.54** | **3.8e-62** | 0.08 | ns | 0.1 | ns | 0.04 | ns | 0.08 | ns | **0.18** | **3.3e-04** | **0.18** | **1.3e-04** | **0.16** | **7.8e-03** |
| ALT | 0.09 | ns | **0.49** | **8.4e-49** | **0.31** | **2.2e-16** | **0.42** | **2.7e-33** | **0.17** | **2.4e-03** | 0.07 | ns | -0.02 | ns | 0.13 | ns | **0.17** | **1.1e-03** | **0.16** | **6.6e-03** | **0.15** | **1.3e-02** |
| ALP | 1 |  | **0.2** | **4.1e-06** | 0.04 | ns | **-0.16** | **4.0e-03** | 0.06 | ns | 0.05 | ns | -0.08 | ns | -0.06 | ns | -0.09 | ns | **0.17** | **5.6e-04** | -0.01 | ns |
| GGT | **0.2** | **4.1e-06** | 1 |  | **0.16** | **7.8e-03** | 0.13 | ns | **0.17** | **2.3e-03** | 0.02 | ns | 0.06 | ns | **0.21** | **2.0e-06** | **0.17** | **1.6e-03** | 0.13 | ns | 0.10 | ns |
| LDH | 0.04 | ns | **0.16** | **7.8e-03** | 1 |  | **0.33** | **7.4e-19** | **0.17** | **2.9e-03** | 0.08 | ns | -0.06 | ns | 0 | ns | 0.06 | ns | **0.17** | **1.7e-03** | 0.10 | ns |
| CK | **-0.16** | **4.0e-03** | 0.13 | ns | **0.33** | **7.4e-19** | 1 |  | **0.18** | **5.7e-04** | 0.03 | ns | 0.06 | ns | **0.19** | **5.6e-05** | 0.08 | ns | 0.1 | ns | **0.20** | **1.4e-05** |
| CHE | 0.06 | ns | **0.17** | **2.3e-03** | **0.17** | **2.9e-03** | **0.18** | **5.7e-04** | 1 |  | 0.04 | ns | 0.01 | ns | **0.18** | **2.4e-04** | -0.11 | ns | **0.29** | **4.7e-14** | **0.35** | **4.8e-21** |
| urea | 0.05 | ns | 0.02 | ns | 0.08 | ns | 0.03 | ns | 0.04 | ns | 1 |  | **0.28** | **1.5e-13** | **0.16** | **5.7e-03** | -0.04 | ns | 0.08 | ns | 0.07 | ns |
| creatinine | -0.08 | ns | 0.06 | ns | -0.06 | ns | 0.06 | ns | 0.01 | ns | **0.28** | **1.5e-13** | 1 |  | **0.43** | **1.0e-35** | 0.1 | ns | 0.05 | ns | 0.13 | ns |
| Uric acid | -0.06 | ns | **0.21** | **2.0e-06** | 0 | ns | **0.19** | **5.6e-05** | **0.18** | **2.4e-04** | **0.16** | **5.7e-03** | **0.43** | **1.0e-35** | 1 |  | 0.1 | ns | 0.07 | ns | 0.08 | ns |
| Bilirubin | -0.09 | ns | **0.17** | **1.6e-03** | 0.06 | ns | 0.08 | ns | -0.11 | ns | -0.04 | ns | 0.1 | ns | 0.1 | ns | 1 |  | -0.01 | ns | 0.01 | ns |
| Tot. prot. | **0.17** | **5.6e-04** | 0.13 | ns | **0.17** | **1.7e-03** | 0.1 | ns | **0.29** | **4.7e-14** | 0.08 | ns | 0.05 | ns | 0.07 | ns | -0.01 | ns | 1 |  | **0.77** | **2.2e-158** |
| Albumin | -0.01 | ns | 0.10 | ns | 0.10 | ns | **0.20** | **1.4e-05** | **0.35** | **4.8e-21** | 0.07 | ns | 0.13 | ns | 0.08 | ns | 0.01 | ns | **0.77** | **2.2e-158** | 1 |  |
| CHO | 0.04 | ns | **0.2** | **9.9e-06** | **0.23** | **2.4e-08** | 0.09 | ns | **0.35** | **6.2e-22** | 0.02 | ns | -0.06 | ns | 0.06 | ns | -0.09 | ns | **0.29** | **2.9e-14** | **0.34** | **2.3e-20** |
| HDL | 0.03 | ns | -0.1 | ns | 0.12 | ns | -0.08 | ns | -0.07 | ns | -0.01 | ns | -0.12 | ns | **-0.32** | **7.4e-18** | -0.01 | ns | 0.14 | ns | **0.15** | **2.4e-02** |
| LDL | 0.02 | ns | **0.16** | **2.4e-03** | 0.08 | ns | 0.09 | ns | **0.33** | **5.0e-19** | 0.01 | ns | -0.02 | ns | 0.13 | ns | -0.08 | ns | **0.23** | **2.4e-08** | **0.28** | **1.2e-13** |
| TG | 0.06 | ns | **0.27** | **2.3e-12** | 0.06 | ns | 0.12 | ns | **0.32** | **5.3e-17** | 0.05 | ns | 0.09 | ns | **0.36** | **1.2e-23** | -0.06 | ns | 0.11 | ns | 0.13 | ns |
| Na^+^ | -0.02 | ns | -0.08 | ns | 0.02 | ns | -0.02 | ns | 0 | ns | 0.06 | ns | 0 | ns | -0.03 | ns | 0.04 | ns | -0.12 | ns | -0.07 | ns |
| K^+^ | 0.02 | ns | 0 | ns | -0.03 | ns | -0.04 | ns | 0 | ns | 0.1 | ns | 0.12 | ns | 0.03 | ns | -0.04 | ns | **0.15** | **8.6e-03** | **0.15** | **2.3e-02** |
| Cl^-^ | -0.09 | ns | -0.08 | ns | **0.27** | **2.9e-12** | 0 | ns | -0.01 | ns | 0.02 | ns | 0 | ns | -0.04 | ns | 0.02 | ns | **-0.27** | **4.2e-12** | **-0.24** | **1.4e-09** |
| Ca^2+^ | 0.06 | ns | 0.09 | ns | **-0.18** | **2.3e-04** | 0 | ns | **0.2** | **7.6e-06** | 0.03 | ns | -0.01 | ns | 0.06 | ns | 0 | ns | **0.45** | **9.1e-39** | **0.43** | **3.2e-34** |
| CRP | **0.26** | **2.9e-07** | **0.18** | **2.1e-02** | 0.09 | ns | 0.01 | ns | 0.09 | ns | 0.03 | ns | -0.03 | ns | 0.15 | ns | -0.07 | ns | 0.07 | ns | -0.14 | ns |
| glucose | 0.08 | ns | 0.1 | ns | -0.03 | ns | **0.16** | **6.6e-03** | **0.16** | **3.7e-03** | 0.1 | ns | 0.08 | ns | **0.21** | **3.2e-06** | -0.04 | ns | **0.26** | **3.6e-11** | **0.25** | **1.5e-10** |
| TSH | 0.01 | ns | 0.07 | ns | 0.04 | ns | 0.05 | ns | 0.03 | ns | -0.01 | ns | 0.07 | ns | 0.05 | ns | -0.03 | ns | 0.05 | ns | 0.10 | ns |

**Supplementary Table S6 *(cont.)***

|  | **CHO** | | **HDL** | | **LDL** | | **TG** | | **Na^+^** | | **K^+^** | | **Cl^-^** | | **Ca^2+^** | | **CRP** | | **Glucose** | | **TSH** | |
| --- | --- | --- | --- | --- | --- | --- | --- | --- | --- | --- | --- | --- | --- | --- | --- | --- | --- | --- | --- | --- | --- | --- |
|  | **rho** | ***p*** | **rho** | ***p*** | **rho** | ***p*** | **rho** | ***p*** | **rho** | ***p*** | **rho** | ***p*** | **rho** | ***p*** | **rho** | ***p*** | **rho** | ***p*** | **rho** | ***p*** | **rho** | ***p*** |
| RBC | **0.15** | **2.9e-02** | **-0.16** | **2.7e-03** | **0.19** | **2.0e-05** | **0.2** | **4.2e-06** | 0.01 | ns | 0.1 | ns | **-0.17** | **6.2e-04** | **0.21** | **1.5e-06** | 0.07 | ns | **0.17** | **6.3e-04** | 0.03 | ns |
| Hb | **0.18** | **1.2e-04** | **-0.15** | **3.2e-02** | **0.2** | **1.4e-05** | **0.26** | **4.6e-11** | -0.01 | ns | 0.1 | ns | **-0.16** | **2.8e-03** | **0.15** | **3.0e-02** | 0.01 | ns | **0.19** | **5.2e-05** | 0.06 | ns |
| PLT | **0.2** | **3.5e-06** | **0.16** | **8.1e-03** | 0.13 | ns | 0.03 | ns | -0.09 | ns | 0.09 | ns | -0.11 | ns | **0.19** | **1.4e-04** | 0.09 | ns | 0.11 | ns | -0.03 | ns |
| PMN | 0.07 | ns | -0.06 | ns | 0.06 | ns | **0.16** | **5.0e-03** | -0.06 | ns | 0.08 | ns | **-0.2** | **8.2e-06** | **0.19** | **9.3e-05** | **0.31** | **2.9e-11** | **0.25** | **3.0e-10** | 0.01 | ns |
| Ly | 0.13 | ns | -0.13 | ns | 0.13 | ns | **0.16** | **3.0e-03** | 0 | ns | -0.03 | ns | 0.07 | ns | -0.03 | ns | 0 | ns | -0.11 | ns | 0.08 | ns |
| Mo | -0.05 | ns | -0.09 | ns | -0.02 | ns | 0.06 | ns | -0.04 | ns | 0.1 | ns | -0.14 | ns | 0.03 | ns | **0.23** | **1.7e-05** | 0.08 | ns | 0.02 | ns |
| B12 | 0.1 | ns | 0.13 | ns | 0.04 | ns | -0.04 | ns | -0.08 | ns | 0.04 | ns | -0.09 | ns | 0.12 | ns | 0.05 | ns | -0.01 | ns | 0.01 | ns |
| ESR | 0.11 | ns | -0.03 | ns | 0.1 | ns | 0.09 | ns | -0.11 | ns | -0.01 | ns | -0.09 | ns | 0.04 | ns | **0.37** | **1.7e-17** | 0.09 | ns | -0.01 | ns |
| AST | 0.1 | ns | 0.08 | ns | 0.03 | ns | 0.08 | ns | -0.06 | ns | 0.01 | ns | -0.06 | ns | 0.06 | ns | 0.02 | ns | 0.06 | ns | 0.09 | ns |
| ALT | 0.13 | ns | -0.07 | ns | 0.08 | ns | **0.21** | **5.8e-07** | -0.07 | ns | 0 | ns | -0.06 | ns | 0.08 | ns | 0.06 | ns | 0.09 | ns | 0.07 | ns |
| ALP | 0.04 | ns | 0.03 | ns | 0.02 | ns | 0.06 | ns | -0.02 | ns | 0.02 | ns | -0.09 | ns | 0.06 | ns | **0.26** | **2.9e-07** | 0.08 | ns | 0.01 | ns |
| GGT | **0.2** | **9.9e-06** | -0.1 | ns | **0.16** | **2.4e-03** | **0.27** | **2.3e-12** | -0.08 | ns | 0 | ns | -0.08 | ns | 0.09 | ns | **0.18** | **2.1e-02** | 0.1 | ns | 0.07 | ns |
| LDH | **0.23** | **2.4e-08** | 0.12 | ns | 0.08 | ns | 0.06 | ns | 0.02 | ns | -0.03 | ns | **0.27** | **2.9e-12** | **-0.18** | **2.3e-04** | 0.09 | ns | -0.03 | ns | 0.04 | ns |
| CK | 0.09 | ns | -0.08 | ns | 0.09 | ns | 0.12 | ns | -0.02 | ns | -0.04 | ns | 0 | ns | 0 | ns | 0.01 | ns | **0.16** | **6.6e-03** | 0.05 | ns |
| CHE | **0.35** | **6.2e-22** | -0.07 | ns | **0.33** | **5.0e-19** | **0.32** | **5.3e-17** | 0 | ns | 0 | ns | -0.01 | ns | **0.2** | **7.6e-06** | 0.09 | ns | **0.16** | **3.7e-03** | 0.03 | ns |
| urea | 0.02 | ns | -0.01 | ns | 0.01 | ns | 0.05 | ns | 0.06 | ns | 0.1 | ns | 0.02 | ns | 0.03 | ns | 0.03 | ns | 0.1 | ns | -0.01 | ns |
| creatinine | -0.06 | ns | -0.12 | ns | -0.02 | ns | 0.09 | ns | 0 | ns | 0.12 | ns | 0 | ns | -0.01 | ns | -0.03 | ns | 0.08 | ns | 0.07 | ns |
| Uric acid | 0.06 | ns | **-0.32** | **7.4e-18** | 0.13 | ns | **0.36** | **1.2e-23** | -0.03 | ns | 0.03 | ns | -0.04 | ns | 0.06 | ns | 0.15 | ns | **0.21** | **3.2e-06** | 0.05 | ns |
| Bilirubin | -0.09 | ns | -0.01 | ns | -0.08 | ns | -0.06 | ns | 0.04 | ns | -0.04 | ns | 0.02 | ns | 0 | ns | -0.07 | ns | -0.04 | ns | -0.03 | ns |
| Tot. prot. | **0.29** | **2.9e-14** | 0.14 | ns | **0.23** | **2.4e-08** | 0.11 | ns | -0.12 | ns | **0.15** | **8.6e-03** | **-0.27** | **4.2e-12** | **0.45** | **9.1e-39** | 0.07 | ns | **0.26** | **3.6e-11** | 0.05 | ns |
| Albumin | **0.34** | **2.3e-20** | **0.15** | **0.02** | **0.28** | **1.2e-13** | 0.13 | ns | -0.07 | ns | **0.15** | **0.02** | **-0.24** | **1.4e-09** | **0.43** | **3.2e-34** | -0.14 | ns | **0.25** | **1.5e-10** | 0.10 | ns |
| CHO | 1 |  | **0.19** | **9.4e-05** | **0.91** | **0.0e+00** | **0.38** | **1.3e-26** | -0.06 | ns | 0.04 | ns | -0.12 | ns | **0.21** | **1.2e-06** | 0.07 | ns | 0.06 | ns | 0.05 | ns |
| HDL | **0.19** | **9.4e-05** | 1 |  | -0.08 | ns | **-0.51** | **4.4e-53** | 0.05 | ns | 0.05 | ns | -0.08 | ns | **0.16** | **5.8e-03** | **-0.19** | **2.9e-03** | -0.03 | ns | -0.01 | ns |
| LDL | **0.91** | **0.0e+00** | -0.08 | ns | 1 |  | **0.4** | **9.4e-31** | -0.08 | ns | 0.06 | ns | **-0.15** | **2.7e-02** | **0.18** | **2.7e-04** | 0.13 | ns | 0.08 | ns | 0.04 | ns |
| TG | **0.38** | **1.3e-26** | **-0.51** | **4.4e-53** | **0.4** | **9.4e-31** | 1 |  | -0.08 | ns | -0.02 | ns | -0.05 | ns | 0.11 | ns | 0.14 | ns | 0.13 | ns | 0.07 | ns |
| Na^+^ | -0.06 | ns | 0.05 | ns | -0.08 | ns | -0.08 | ns | 1 |  | -0.03 | ns | **0.38** | **2.2e-27** | -0.06 | ns | 0.06 | ns | -0.06 | ns | -0.02 | ns |
| K^+^ | 0.04 | ns | 0.05 | ns | 0.06 | ns | -0.02 | ns | -0.03 | ns | 1 |  | -0.06 | ns | 0.11 | ns | 0.06 | ns | 0.02 | ns | -0.07 | ns |
| Cl^-^ | -0.12 | ns | -0.08 | ns | **-0.15** | **2.7e-02** | -0.05 | ns | **0.38** | **2.2e-27** | -0.06 | ns | 1 |  | **-0.31** | **5.4e-16** | -0.07 | ns | **-0.2** | **6.0e-06** | -0.04 | ns |
| Ca^2+^ | **0.21** | **1.2E-06** | **0.16** | **5.8e-03** | **0.18** | **2.7e-04** | 0.11 | ns | -0.06 | ns | 0.11 | ns | **-0.31** | **5.4e-16** | 1 |  | -0.02 | ns | **0.22** | **8.5e-07** | 0.04 | ns |
| CRP | 0.07 | ns | **-0.19** | **2.9e-03** | 0.13 | ns | 0.14 | ns | 0.06 | ns | 0.06 | ns | -0.07 | ns | -0.02 | ns | 1 |  | 0.08 | ns | 0.07 | ns |
| glucose | 0.06 | ns | -0.03 | ns | 0.08 | ns | 0.13 | ns | -0.06 | ns | 0.02 | ns | **-0.2** | **6.0e-06** | **0.22** | **8.5e-07** | 0.08 | ns | 1 |  | 0.01 | ns |
| TSH | 0.05 | ns | -0.01 | ns | 0.04 | ns | 0.07 | ns | -0.02 | ns | -0.07 | ns | -0.04 | ns | 0.04 | ns | 0.07 | ns | 0.01 | ns | 1 |  |
